# Comparative gene expression profiling of human metallothionein-3 up-regulation in neuroblastoma cells and its impact on susceptibility to cisplatin

**DOI:** 10.18632/oncotarget.23333

**Published:** 2017-12-16

**Authors:** Miguel Angel Merlos Rodrigo, Simona Dostalova, Hana Buchtelova, Vladislav Strmiska, Petr Michalek, Sona Krizkova, Ales Vicha, Pavla Jencova, Tomas Eckschlager, Marie Stiborova, Zbynek Heger, Vojtech Adam

**Affiliations:** ^1^ Department of Chemistry and Biochemistry, Mendel University in Brno, CZ-613 00 Brno, Czech Republic; ^2^ Central European Institute of Technology, Brno University of Technology, CZ-616 00 Brno, Czech Republic; ^3^ Department of Paediatric Haematology and Oncology, 2nd Faculty of Medicine, Charles University, and University Hospital Motol, CZ-150 06 Prague 5, Czech Republic; ^4^ Department of Biochemistry, Faculty of Science, Charles University, CZ-128 40 Prague 2, Czech Republic

**Keywords:** apoptosis, cisplatin, chemoresistance, metallothionein, oncogene-induced senescence

## Abstract

Human metallothionein-3 (hMT-3), also known as growth inhibitory factor, is predominantly expressed in the central nervous system. hMT-3 is presumed to participate in the processes of heavy metal detoxification, regulation of metabolism and protection against oxidative damage of free radicals in the central nervous system; thus, it could play important neuromodulatory and neuroprotective roles. However, the primary functions of hMT-3 and the mechanism underlying its multiple functions in neuroblastoma have not been elucidated so far. First, we confirmed relatively high expression of hMT-3 encoding mRNA in biopsies (*n* = 23) from high-risk neuroblastoma subjects. Therefore, we focused on investigation of the impact of hMT-3 up-regulation in *N-Myc* amplifying neuroblastoma cells. The differentially up-regulated genes involved in biological pathways related to cellular senescence and cell cycle were identified using electrochemical microarray with consequent bioinformatic processing. Further, as experimental verification of microarray data, the cytotoxicity of the cisplatin (CDDP) was examined in hMT-3 and mock cells by MTT and clonogenic assays. Overall, our data strongly suggest that up-regulation of hMT-3 positively correlates with the genes involved in oncogene-induced senescence (*CDKN2B* and *ANAPC5*) or apoptosis (*CASP4*). Moreover, we identified a significant increase in chemoresistance to cisplatin (CDDP) due to hMT-3 up-regulation (24IC_50_: 7.5 *vs*. 19.8 μg/ml), indicating its multipurpose biological significance.

## INTRODUCTION

Metallothionein (MT) family is a class of low molecular mass, intracellular, cysteine-rich proteins with a high affinity to metals. Total MTs were firstly isolated from horse kidney and characterized by Margoshes and Vallee [1]. All vertebrates examined contain at least two or more distinct MT isoforms designated MT-1 through MT-4. MT-3 was originally dubbed neuronal growth-inhibitory factor (GIF) due to its neuroinhibitory activity [2]. Besides, MT-3 might also participate in the processes of metal detoxification, metabolism regulation, and protection from damage caused by oxidative free radicals in central nervous system (CNS) [3]. In the past few years, MT-3 had been postulated to be a multipurpose protein, which could play important neuromodulatory and neuroprotective roles in CNS besides the common roles of MTs [4], including those connected with neurodegenerative diseases [5]. MT-3 shows a brain-specific expression, mainly in glutamatergic neurons; however, up-regulation of these proteins has also been found in a number of cancers, where its presence positively correlates with the poor survival prognosis [6].

Neuroblastoma (Nbl) is a malignancy of the sympathetic ganglia and adrenal medulla, structures derived from the embryonic neural crest. It is the most common extracranial solid cancer in children younger than 5-years and the most common cancer in infants [7–10]. Although, the connection between CNS cancers and MT-3 was already described (e.g. up-regulation and poor survival for glioblastoma multiforme [11]), the primary function of MT-3 and the mechanisms underlying its effect on gene expression in Nbl were not elucidated so far.

Cisplatin or *cis*-diamminedichloroplatinum (CDDP) is one of the most commonly used drugs in the treatment of Nbl [12], inducing cytotoxic cell death mediated by activation of death receptor-mediated apoptotic signaling mechanisms as well as mitochondrial pathways [13, 14]. This reactive drug interacts not only with DNA but also with proteins. One of the current accepted opinions states that damage to various cytoplasmic proteins is an early process that initiates CDDP-induced apoptosis [15, 16]. It is worth noting that the concentration of MTs increases in the moment of administration of the platinum-based drugs. Such stimulated MTs can also rapidly bind the administered CDDP, which can result in decrease of drug concentration below the effective level [17, 18].

To date, as far as we are aware, no information concerning the complex role of MT-3 in Nbl cells exists. Therefore, to unravel the putative mechanisms involved in influencing of enhanced expression of human MT-3 (hMT-3) in Nbl cells, we carried out comparative screening of gene expression by cDNA microarray. We utilized SiMa cell line derived from high-risk Nbl with *N-Myc* amplification and loss of chromosome 11, which is a well-characterized model of human neuronal growth and differentiation [19]. To increase the expression of hMT-3 we transiently transfected SiMa cells with a plasmid containing *hMT-3* gene (pcDNA3.1-GFP-hMT-3-TOPO) or with an empty vector (pcDNA3.1-GFP-TOPO). The main aim was to promote novel insights into the molecular mechanisms of hMT-3 up-regulation and to elucidate the effects beneath the hMT-3 up-regulation in Nbl cells. Hence, we performed comparative microarray survey with a special emphasis on expression of genes driving pivotal cancer-related molecular pathways. Moreover, we also focused on experimental verification of the hypothesis that hMT-3 is able to increase chemoresistance of Nbl cells to CDDP.

## RESULTS

### hMT-3 expression in Nbl biopsies and non-malignant cell lines derived from adrenal cortex

In order to verify that Nbl expresses hMT-3, we examined 23 high-risk (HR) Nbl specimens and quantified hMT-3 expression. Patient data are shown in Supplementary Table 1. All samples expressed hMT-3 within the range of 2^-ΔΔ^Ct 12.550–19.245. Although we did not show any significant relationship to the prognosis, amplification of *N-Myc*, or whether a sample was taken before or during chemotherapy, it is worth to note that high-risk Nbl express relatively high amount of mRNA encoding hMT-3. As Nbl mostly starts from one of the adrenal glands, we further investigated hMT-3 expression in non-malignant cell lines derived from adrenal cortex. Noteworthy, we found that normal adrenal cortex-derived cells express mRNA encoding hMT-3, which is in good agreement with study by Felizola *et al*. [20]. However, the 2^-ΔΔ^Ct values were considerably lower (0.699–2.365) compared with HR Nbl specimens. Overall, these findings underpin an importance of hMT-3 in Nbl. This prompted us to continue with isolation and cloning of hMT-3 for consequent transfections, followed by microarray survey and phenotypic analyses.

### Transfection of SiMA cells with pcDNA3.1-GFP-hMT-3-TOPO (hMT-3) or pcDNA3.1-GFP-TOPO (mock)

**Figure 1:**
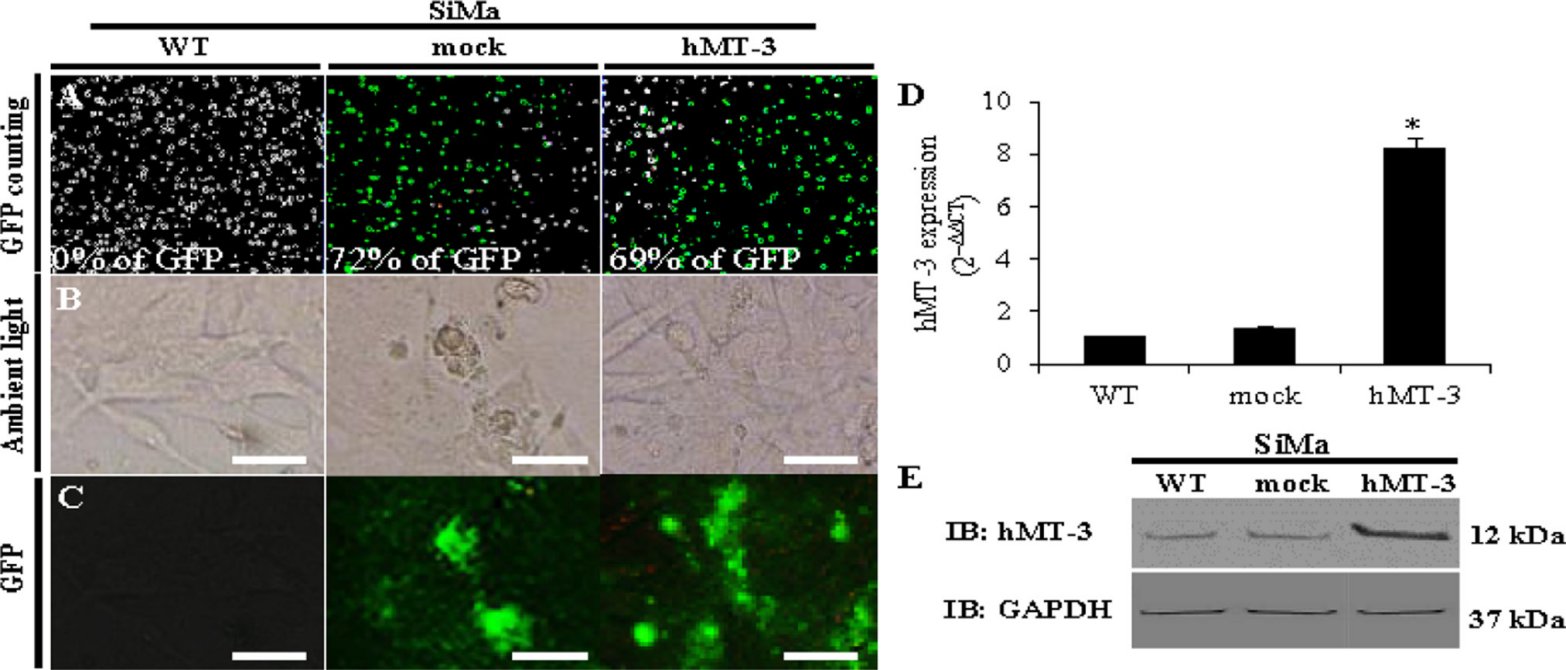
Comparison of WT, mock and hMT-3 neuroblastoma (SiMa) cells The cells were transfected with either pcDNA3.1-GFP-TOPO (mock transfection) or pcDNA3.1-GFP-hMT3-TOPO (hMT-3). Transfection efficiency and cells viability was estimated by (**A**) fluorescence cell counting, (**B**) ambient and (**C**) fluorescence microscopy. For comparison, non-transfected - wild-type (WT) cells are included. The length of scale bar is 30 μm. (**D**) qRT-PCR showing changes in mRNA encoding hMT-3. Data were analyzed by comparative C_T_ method and presented as relative fold gene expression (2^−ΔΔCT^). (**E**) Representative immunoblots analyses of whole-cell lysates evaluated for hMT-3 expression. GAPDH served as loading control. Data with asterisk (^*^) indicate statistical significance (*p* < 0.05).

Figure 1A demonstrates the efficiency of transfection analysed through a fluorescence of GFP tag expressed at the *C*-terminus of hMT-3. The results show that our optimized transfection protocol resulted in approx. 70% transfection efficiency for both constructed plasmids (mock and hMT-3). An ambient and fluorescence microscopy revealed formation of large bright-green GFP aggregates in mock cells and in lesser content also in hMT-3 counterparts (Figure 1B and Figure 1C).

Further, the qRT-PCR confirmed significant (*p* < 0.05) increase in the expression of hMT-3 (Figure 1D). In this case, 2^−ΔΔCT^ method revealed that the transfection with hMT-3 resulted in 8-fold higher relative expression compared with WT SiMa cells or mock cultures. Finally, Western blotting with rabbit anti-MT-3 antibody confirmed pronouncedly increased expression of hMT-3 in the hMT-3 transfected SiMa extract, while mock transfection showed comparable hMT-3 expression to that of WT cells as shown in Figure 1E.

### hMT-3 up-regulation in SiMa cells influences expression of genes involved in oncogene-induced senescence (OIS) and cell cycle

**Figure 2:**
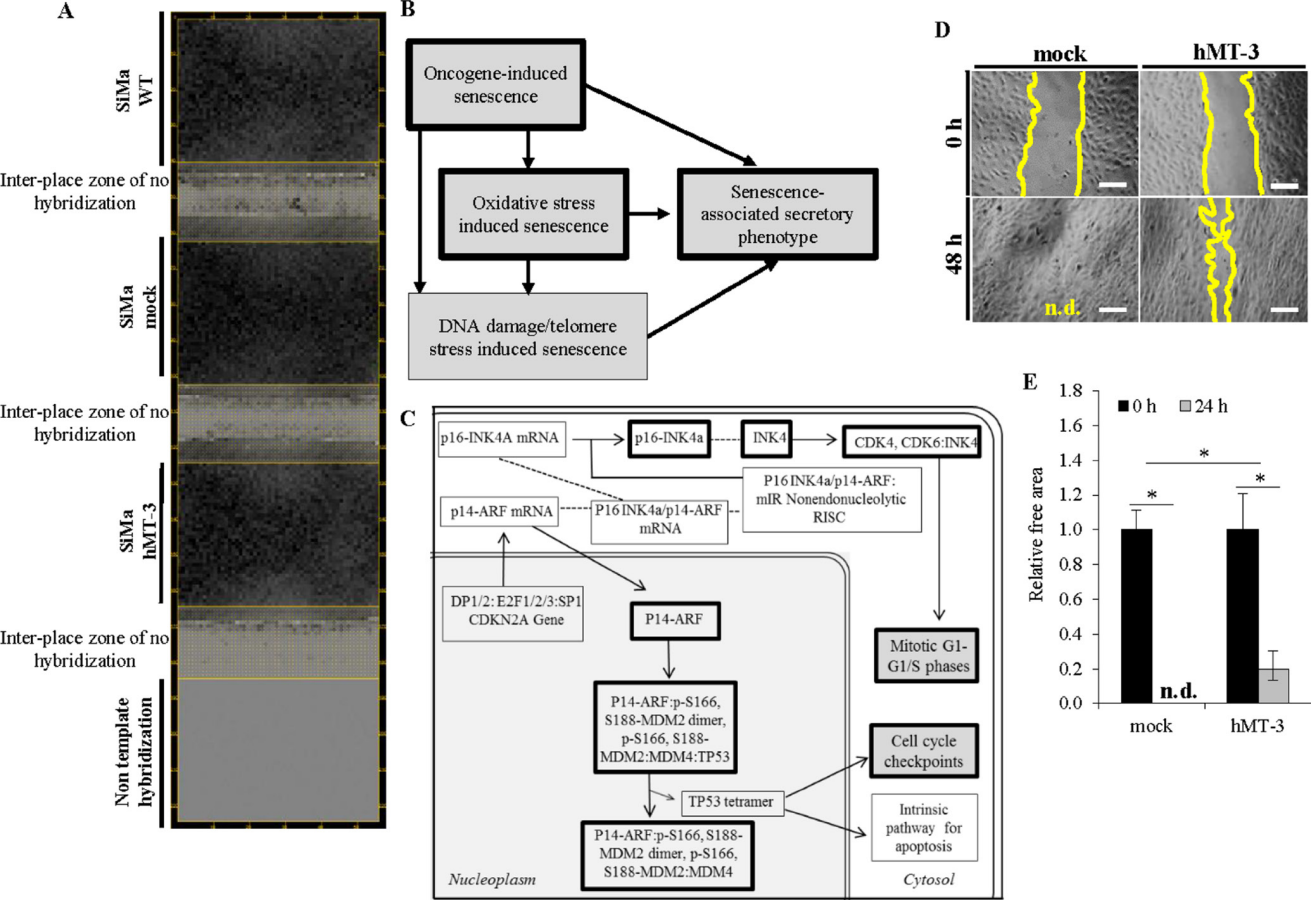
Comparative bioinformatical processing of microarray data (**A**) Representative microarray heatmaps showing gene expressions in SiMa cells (one spot per one gene). Gray scale intensity represents the rate of individual mRNA expression. (**B**) hMT-3 induced over-expression of genes was identified to affect the regulation of cellular senescence pathway (data were analyzed by Reactome, http://www.reactome.org/). (**C**) Schematic drawing of oncogene-induced senescence pathway. Black framings indicate genes identified as up-regulated after hMT-3 transfection. (**D**) Representative micrographs of wound-healing assay showing slower migration of hMT-3 cells. Micrographs demonstrate the artificial wounds at the experimental start-point (0 h) and the migration of the cells after 48 h incubation. The length of scale bar is 100 μm, n.d. not detected. (**E**) Quantitation of relative free areas. The values are expressed as the mean of six independent replicates (*n* **=** 6). Data with asterisks (^*^) indicate statistical significance (*p* < 0.05).

We investigated the cancer-related genes affected by hMT-3 up-regulation using electrochemical microarray (expression heatmap is shown in Figure 2A). Table 1 shows a list of genes, along with their accession numbers, which were found up- and down-regulated in three independent analyses (*n* = 3, the genes with Fold ratio > 1.5, which were considered as significantly up-regulated, are displayed only). Our analyses revealed that hMT-3 up-regulation induced up- or down-regulation of several genes (20 *vs*. 3, respectively). To confirm selected microarray results we separately performed semiquantitative (SQ) RT-PCR of five selected genes with the highest expression (*CSPG2*, *ANAPC5*, *PIAS2*, *BMP1* and *ASPM*) and *MT-3*. To adjust the amount of transcribed cDNA, *18S rRNA* was selected as an internal control. Validation of microarrays by SQ-RT-PCR for selected genes is shown in Supplementary Figure 1.

**Table 1:** List of genes up- or down-regulated after transfection with hMT-3 or in mock culture

hMT-3 *vs.* mock				
Up-regulation				
Gene	Symbol	NCBI database	Fold ratio	SD (*n* = 3)
Chondroitin sulfate proteoglycan 2	*CSPG2*	NM_004385	2.947	3.451
Anaphase promoting complex subunit 5	*ANAPC5*	NM_016237	2.670	0.201
Protein inhibitor of activated STAT, 2	*PIAS2*	NM_173206	2.089	0.537
Bone morphogenetic protein 1	*BMP1*	NM_001199	1.872	0.438
Asp (abnormal spindle)-like, microcephaly associated	*ASPM*	NM_018136	1.804	0.145
L-3-hydroxyacyl-Coenzyme A dehydrogenase, short chain	*HADHSC*	NM_005327	1.672	0.201
Cyclin-dependent kinase inhibitor 2B (p15, inhibits CDK4)	*CDKN2B*	NM_004936	1.653	0.064
Glutathione S-transferase M3	*GSTM3*	NM_000849	1.646	0.220
Centromere protein A, 17kDa	*CENPA*	NM_001809	1.605	0.288
Caspase 4, apoptosis-related cysteine peptidase	*CASP4*	NM_001225	1.586	0.045
Hypothetical protein FLJ12443	*FLJ12443*	NM_024830	1.582	0.229
Small nuclear ribonucleoprotein polypeptides B and B1	*SNRPB*	NM_003091	1.578	0.426
Plasminogen activator, tissue	*PLAT*	NM_033011	1.544	0.004
DnaJ (Hsp40) homolog, subfamily B, member 6	*DNAJB6*	NM_058246	1.542	0.137
DEAD (Asp-Glu-Ala-Asp) box polypeptide 21	*DDX21*	NM_004728	1.540	0.147
Pre-B-cell colony enhancing factor 1	*PBEF1*	NM_005746	1.538	0.075
Lactate dehydrogenase B	*LDHB*	NM_002300	1.524	0.497
Sema domain, immunoglobulin domain (Ig), short basic domain, secreted, (semaphorin) 3B	*SEMA3B*	NM_001005914	1.523	0.026
Metallothionein 3	*MT3*	NM_005946	1.520	0.018
Palmitoyl-protein thioesterase 1 (ceroid-lipofuscinosis, neuronal 1, infantile)	*PPT1*	NM_000310	1.504	0.016
**Down-regulation**				
**Gene**	**Symbol**	**NCBI database**	**Fold ratio**	**SD (*n*** **= 3)**
Histone deacetylase 2	*HDAC2*	NM_001527	0.470	0.315
X-ray repair complementing defective repair 3	*XRCC3*	NM_005432	0.458	0.162
CD47 antigen (Rh-related antigen, integrin-associated signal transducer)	*CD47*	NM_001025079	0.114	0.0109

We further carried out the gene ontology (GO) analysis of involvement of the up-regulated genes within biological pathways related to regulation of biological functions, response to stimuli and homeostatic processes. Table 2 shows the list of processes and/or pathways of gene regulation in hMT-3 SiMa cells using GO annotations and KEGG 10 software, respectively. Noteworthy, the bioinformatic analyses revealed the up-regulation of numerous genes affecting biological pathways related to cellular senescence (schematized in Figure 2B) and OIS (Figure 2C). The OIS is a robust and sustained antiproliferative response brought by oncogenic signaling resulting from an activating mutation of an oncogene, or the inactivation of a tumor-suppressor gene [21–23]. We identified two major targets: i) *CDKN2B* (cyclin dependent kinase inhibitor 2B) and ii) *ANAPC5* (anaphase promoting complex subunit 5), which belong to biological pathways related to OIS [24–26]. We also identified increase in glutathione *S*-transferase M3 (*GSTM3*), which is a member of the superfamily of GST enzymes and which may correspond to the initiation of self-protective machinery of cells in tumorigenetic process [27].

**Table 2:** The list of processes and/or pathways involved in gene regulation in SiMa cells (hMT-3 vs. mock) using gene ontology (GO) annotations and KEGG 10 software

hMT-3 *vs.* mock			
Up-regulation			
pathway ID	pathway description	observed gene count	false discovery rate
GO.0007094	mitotic spindle assembly checkpoint	5	1.07E-06
GO.0070979	protein K11-linked ubiquitination	5	1.07E-06
GO.0007093	mitotic cell cycle checkpoint	6	2.32E-05
GO.0009896	positive regulation of catabolic process	8	2.35E-05
GO.0031145	anaphase-promoting complex-dependent proteasomal ubiquitin-dependent protein catabolic process	5	2.35E-05
GO.0045861	negative regulation of proteolysis	7	4.65E-05
GO.1903047	mitotic cell cycle process	8	0.0003
GO.0000278	mitotic cell cycle	8	0.0006
**Down-regulation**			
**pathway ID**	**pathway description**	**observed gene count**	**false discovery rate**
GO:0007155	ECM-receptor interaction	1	0.0139
GO:0043044	Notch signaling pathway	1	0.0012

Among others, *CASP4* (caspase-4) plays role in sequential activation of caspases, which is a central role in the execution-phase of cell apoptosis [28]. *DNAJB6* [DnaJ heat shock protein family (Hsp40) member B6] is involved in a wide range of cellular events, such as protein folding and oligomeric protein complex assembly and has a relevant functional role in neurons [29].

We also performed analysis of hMT-3 effect on migration of hMT-3 *vs.* mock cultures. Figure 2D and 2E illustrate that hMT-3 up-regulation significantly (*p* < 0.05) decreased the migration of transfected cells.

Overall, we show that hMT-3 has a significant biological role affecting regulatory pathways, which could pronouncedly influence the susceptibility of cancer cells to chemotherapy. It must be mentioned that our results are based on gene expression, which can be biased due to various aspects of cellular biology (e.g. by epigenetic silencing and subsequent translational repressions). Hence, we also focused on experimental verification of obtained results in terms of evaluation of impact of hMT-3 up-regulation on cytotoxic effects of CDDP.

### hMT-3 causes pronounced resistance to cytotoxic activity of CDDP

**Figure 3:**
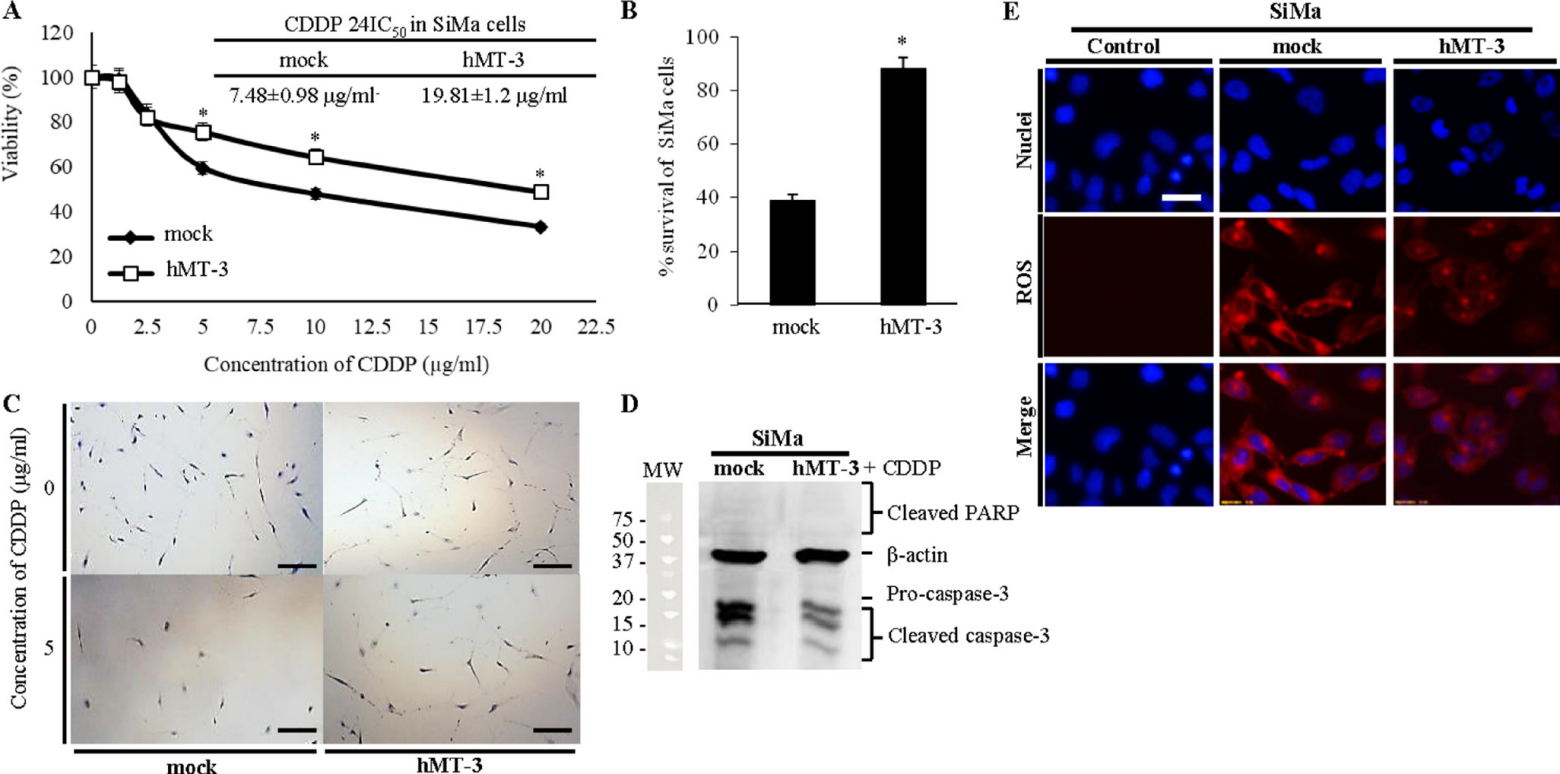
Investigation of hMT-3 up-regulation on cytotoxicity of CDDP (**A**) MTT survival curves of mock and hMT-3 cells exposed to CDDP (1.2–20.0 μg/ml) for 24 h. Inserted are 24IC_50_ values for both tested cell lines. (**B**) Percentage of survival after application of CDDP determined by clonogenic assay. Data with asterisk (^*^) indicate significant differences (*p* < 0.05). (**C**) Detailed representative micrographs of clonogenic assay plates showing significant differences between mock and hMT-3 cells survival and also clonogenicity. The length of scale bar is 200 μm. The cells were stained with 0.1% crystal violet. (**D**) Estimation of apoptotic markers in mock and hMT-3 cultures after CDDP treatment. β-actin served as loading control. (**E**) Living cell microscopy of CDDP-induced ROS (CellROX, red). Nuclei were counterstained with Hoechst 33258. The length of scale bar is 20 μm.

As an experimental verification of microarray data, showing involvement of hMT-3 into OIS and cell cycle regulation, the susceptibility to CDDP was examined using the MTT and clonogenic assays. As shown in Figure 3A, CDDP induced toxic effects in both hMT-3 and mock cultures. Despite that, we found significant differences in cell viability, highlighted by survival curves (Figure 3A) and by calculated 24IC_50_, which were 7.5 μg/ml *vs.* 19.8 μg/ml (mock *vs*. hMT-3, respectively). Due to the fact that MTT reagent can be significantly influenced by assay conditions and estimates more metabolic activity, not necessarily viability, we decided to carry out validation by clonogenic assay, which is a standard technique for studying the effects on the cells survival and proliferation. Figure 3B illustrates that clonogenic assay corroborated our previous results and that hMT-3 significantly influences chemoresistance of SiMa cells against CDDP. Moreover, Figure 3C depicts that hMT-3 cells reached lower initial confluence and had lower proliferative rate, which is in agreement with wound-healing assay (Figure 2D and 2E) and which could be explained by up-regulation of OIS-related genes.

In addition to these data, Figure 3D demonstrates that hMT-3 up-regulation caused pronounced decrease in cleaved caspase-3, which is activated by both extrinsic and intrinsic apoptotic signals. Moreover, Figure 3E demonstrates that up-regulated hMT-3 acts as a scavenger of CDDP-induced intracellular reactive oxygen species (ROS). Overall, these data are in good agreement with lower susceptibility of hMT-3 SiMa cells to CDDP and highlight the importance of hMT-3 in Nbl biology.

## DISCUSSION

In recent studies, MT up-regulation has been linked with the enhanced cell proliferation in human tumors of breast, colon, kidney, liver, lung, nasopharynx, ovary, prostate and testes [30–34]. Dziegiel *et al.* showed that up-regulation of MT in various malignant tumors could be suggested as a potential factor of poor prognosis [35]. Contrary to that, the current studies in the androgen-independent prostate cancer cells demonstrate that the endogenous up-regulation of MT-3 can inhibit cell growth [36]. Nevertheless, to the best of our knowledge there is a lack of information regarding MT-3 role in Nbl cells. In our initial experiments, we found that despite their chemoresistance to CDDP, hMT-3 cells reached full confluence much slower than mock cells. This corroborates well-known fact that MT-3 acts as neuronal growth inhibitory factor [37] and corresponds to the identified up-regulation of OIS-related genes. Moreover, hMT-3 inhibits cell migration as demonstrated by experiments with artificial wounds.

Our microarrays shed some light on the genes involved in inducing senescence in tested Nbl cells with up-regulated *hMT-3*. Organisms with renewable tissues had to evolve mechanisms to prevent the development of cancer. One such mechanism is cellular senescence, which irreversibly arrests the growth of cells at risk for neoplastic transformation [38]. Cellular senescence, a growth-arrest program that limits the lifespan of mammalian cells and prevents unlimited cell proliferation, is attracting considerable attention because of its links to tumor suppression [39, 40].

Cyclin-dependent kinase 4 inhibitor B, also known as multiple tumor suppressor 2 (MTS-2) or p15INK4B, is a protein encoded by the *CDKN2B* gene in humans. *CDKN2B* gene encodes 2 distinct transcript variants: p15 and p10. The *CDKN2B/p15* gene plays a critical role in cell cycle progression and is considered to be a target for tumor inactivation in Nbl cells [41]. Our results showed that *CDKN2B* was up-regulated in hMT-3 cells. A component of the anaphase promoting complex (APC), a cell cycle-regulated E3 ubiquitin ligase controls progression through mitosis and the G_1_ phase of the cell cycle. Park *et al.* showed a negative correlation between APC expression and a high grade with aneuploidy supports a possible linkage between the downregulation of APC and malignant transformation in breast cancer [42]. Now, the regulation of APC in Nbl cells is not sufficiently known. However, our results showed that the anaphase promoting complex subunit 5 (*ANAPC5*) was up-regulated due to hMT-3 transfection.

GST is abundantly expressed in some mammalian tissues, particularly those associated with malignancies. GSTs have been found to have regulatory roles via kinase interactions and subsequent downstream control of cell proliferation, inflammatory responses, apoptosis and senescence. Enough recent evidences suggest that pharmacological inhibition of these enzymes may be useful for the treatment of cancer and other diseases associated with aberrant cell proliferation [43]. GSTs may be associated with resistance to a range of cytotoxic drugs, some of which are commonly used to treat Nbl cells [44]. Since most cancer drugs are not good substrates for GSTs, the question of why cells with acquired drug resistance have such high levels of this isozyme seems perplexing. Moreover, even without drug selection, GSTs can be one of the more prevalent cytosolic proteins in cancer cells. These observations would seem to indicate that GSTP has a diversity of functions in cancer cells, some of which are likely unrelated to the detoxification of chemicals or drugs [45]. The significant increase of GSTM3 level in Nbl cells may correspond to the initiation of self-protective machinery of cells in tumorigenetic process [27, 46].

Our results further revealed that also caspase-4 (*CASP4*) was up-regulated in hMT-3 cells. CASP is a family of endoproteases that provides critical links in cell regulatory networks controlling apoptosis. Dysregulation of caspases underlies human diseases including cancer and inflammatory disorders, and major efforts to design better therapies for these diseases seek to understand how these enzymes work and how they can be controlled [47]. Sequential activation of caspases plays a central role in the execution-phase of cell apoptosis. Yang *et al.* suggested that CASP4 activity is required for Fas-induced cell apoptosis and indicated that CASP4 is a new mediator of NF-κB pro-apoptotic pathway in Nbl cells [28]. Overall, it is worth noting that hMT-3 up-regulation had crucial biological effects *in vitro* and resulted in phenotype with a lower proliferative rate and aggressiveness. This indicates that contrary to other MT subtypes and CNS cancer [11, 48]; hMT-3 should not be connected with worst prognosis or outcome of patients with Nbl. To fully prove this phenomenon, experiments studying the amount of MT-3 in biopsies and sera of Nbl subjects are on the way.

Relapse disease with the emergence of drug resistant tumor cells is a major impediment to the successful treatment of high-risk Nbl patients. The mechanisms responsible for CDDP resistance are several, and contribute to the multifactorial nature of the problem [49]. Based on many clinical studies, it is well known that a resistance to a treatment by cytostatic agents is a crucial complication of anticancer therapy [17]. As in some clinical settings CDDP constitutes the major therapeutic option, the development of chemosensitization strategies constitutes a goal with important clinical implications [50, 51]. In general, multiple mechanisms have been identified for the acquisition of drug resistance by cancer cells, including: inhibition of apoptosis through activation of the PI3-K/AKT pathway and induction of antiapoptotic Bcl-2 family members, loss of p53 function resulting in altered apoptosis induced by platinum-DNA lesions, and up-regulation of ABC family members causing the efflux of CDDP from the cell [52]. Florea *et al.* showed that one of the intracellular mechanisms of acquired resistance to CDDP increases detoxification of drugs by the thiols glutathione and MTs [53]. Once inside the cell, it interacts with MTs that sequester CDDP and remove it from the cell. Therefore, MT may contribute to CDDP resistance. In some cases, the levels of MT are higher in CDDP-resistant cells, but in other cases, the MT levels are unaffected [54]. Our results showed that up-regulated *hMT-3* pronouncedly increased the resistance to CDDP.

Iolascon *et al.* demonstrated, for the first time, that a significant percentage of Nbl cells lack caspase-3 mRNA and protein [55]. Although, our microarray expression data did not show effect of hMT-3 on caspase-3, immunoblotting revealed that cultures with up-regulated hMT-3 expressed significantly decreased amount of cleaved caspase-3 compared with mock cultures. We anticipate that the decrease in cleaved caspase-3 is mostly associated with an enhanced binding of CDDP to hMT-3 structure. Up-regulated hMT-3 also scavenges CDDP-induced ROS as shown in Figure 3E. These mechanisms consequently contribute to inhibition of apoptosis and decreased requirements for pro-caspase-3 cleavage.

The MTT and clonogenic assays results could shed some light on the MT-3 involved in inducing resistance to CDDP in cancer cells. These data strongly suggest that up-regulated hMT-3 potently induces properties of Nbl cells and their chemoresistance to CDDP, revealing great potential for its further investigation by means of prognostic biomarker. We anticipate that increased expression of hMT-3 within tumor mass should inform about worse prognosis and also decreased efficiency of platinum-based chemotherapy. However, further investigations of this phenomena might be done.

## MATERIALS AND METHODS

### Chemicals

All chemicals and reagents were purchased from Sigma-Aldrich (St. Louis, MO, USA) in ACS purity, unless noted otherwise.

### Tumor tissue and blood samples collection

The high-risk Nbl tissue samples obtained from positive biopsies were collected from 23 patients at University hospital Motol, Prague, Czech Republic from January 2003 to December 2013.

Patients’ data are summarized in Supplementary Table 1. Samples were frozen within 1 h after surgery and were stored in liquid nitrogen until RNA was isolated using High pure total-RNA isolation kit (Roche, Basel, Switzerland). Clinical part of this study was approved by the Ethics Committee of University hospital Motol. In case of Nbl specimens, informed consent was obtained from all parents of patients.

### Cell lines and culture conditions

For transfections and microarray experiments, SiMa (*N-Myc* amplified) cell line established from the adrenal tumor tissue resected after treatment from a 20-month-old boy of European origin with stage III Nbl was employed. We further used following cell lines to evaluate the expression of hMT-3 in non-malignant cells: i) HAdCC (human adrenal cortical cells), primary cell line isolated from normal human adrenal cortical tissue, ii) EJG derived from collagenase dissociated bovine adrenal tissue and iii) SBAC which is a non-malignant cell type with fibroblast morphology derived from adrenal cortex. Except for HAdCC, all cell lines were purchased from American Type Culture Collection (Manassas, VA, USA). HAdCC cells were bought from ScienCell (Carlsbad, CA, USA). SiMa cells were cultured in RPMI-1640, GlutaMAX, with 10% heat inactivated fetal bovine serum (FBS), EJG cells were cultured in MEM with 10% FBS and SBAC cells were cultured in Ham´s F12 with fibroblast growth factor (40 ng/ml) and 10% FBS. HAdCC cells were resuscitated, thawed, and cultured to 3–10 passages in MSCM with 10% FBS. All media were supplemented with penicillin (100 U/ml) and streptomycin (0.1 mg/ml) and cells were cultured in a humidified atmosphere containing 5% CO_2_ at 37°C.

### DNA constructs and cell transfection

Isolated human *MT-3* gene (*hMT-3*) was cloned in the pRSET-B vector (Invitrogen, Waltham, MA, USA, Supplementary Figure 2A). The chemical transformation protocol was performed following the instructions of New England Biolabs (Ipswich, MA, USA), using BL21(DE3)pLysS chemically competent *Escherichia coli* as a host. The amplified plasmid was further isolated by using the Qiagen Miniprep Kit (Qiagen, Germantown, MD, USA). Then, the *hMT-3* gene was amplified by Expand High Fidelity PCR System. The PCR product was cloned into NT-GFP fusion TOPO^®^ TA (Invitrogen) expression vector (Supplementary Figure 2B). The chemical transformation protocol was performed following the instructions of New England Biolabs, using BL21(DE3)pLysS as a host to obtain pcDNA3.1-GFP-hMT-3-TOPO. The positive transformants of *hMT-3* were grown in Luria-Bertani broth with 50 μg/ml ampicillin. The orientation of the *hMT-3* sequence within the cloning vector was checked by Sanger sequencing (Promega, Madison, WI, USA) (Supplementary Figure 2C). SiMa cells were transfected with pcDNA3.1-GFP-hMT-3-TOPO encoding full-length hMT-3. An empty pcDNA3.1-GFP-TOPO was used as the mock control. SiMa cells were incubated on 6-well plate: 1 × 10^6^; for 24 h at 37°C. Then, 200 μl of medium containing 3 μg of pcDNA3.1-GFP-hMT-3-TOPO or pcDNA3.1-GFP-TOPO as control (mock transfection) and 3 μg of polyethyleneimine (PEI) was added and the cells were incubated for another 12 h. After incubation, the medium was replaced with new medium and the cells were incubated for another 24 h. Then, the transfection efficiency was monitored by Countess FL II (Thermo Fisher Scientific, Waltham, MA, USA) and fluorescence microscopy (Olympus IX 71S8F-3 (Olympus, Tokyo, Japan) using the fluorescence of green fluorescence protein (GFP).

### Isolation of RNA and reverse transcription (RT)

High pure total-RNA isolation kit (Roche, Basel, Switzerland) was used for isolation of cellular RNA. The medium was removed and samples were washed twice with 5 ml of ice-cold PBS. Cells were scraped off, transferred to clean tubes and centrifuged at 20 800 × g for 5 min at 4°C. After that, lysis buffer was added and RNA isolation was carried out according to manufacturer's instructions. Similarly, RNA from biopsies was isolated using the same kit. Isolated RNA was used for cDNA synthesis. RNA (500 ng) was transcribed using Transcriptor First Strand cDNA Synthesis Kit (Roche) according to manufacturer's instructions. Prepared cDNA (20 μl) was diluted with RNase-free water to a total volume of 100 μl and 5 μl of this solution was employed for qRT-PCR and microarrays.

### qRT-PCR of hMT-3 encoding mRNA

Gene expression was studied by qRT-PCR using the SYBR Green Quantitative RT-PCR Kit and the Mastercycler pro S instrument (Eppendorf, Hamburg, Germany). The specificity of the q-RT-PCR was checked by melting curve analysis and the relative levels of transcription were calculated using the 2^−ΔΔ^CT method. Further, the SQ-RT-PCR was checked on 1% agarose gel electrophoresis. The list of primers for validation of microarray by SQ-RT-PCR is shown in the Supplementary Table 2. SQ-RT-PCR experiments were performed in conditions described in our previous study [56].

### Western blotting

The cells were harvested by trypsinization and then centrifuged at 10 000 rpm for 10 min. The lysis was done on ice with 200 μl of RIPA lysis buffer containing 2 μl of protease inhibitor cocktail. Extracted protein was stored at −80°C until analyzed. Equal amounts of protein were separated using SDS-PAGE and then electroblotted onto a PVDF membrane. The PVDF membrane was blocked with 1% skimmed milk in PBS (37 mM NaCl, 2.7 mM KCl, 1.4 mM NaH_2_PO_4_, 4.3 mM Na_2_HPO_4_, pH 7.4) and then incubated separately with primary antibodies against MT-3 (1:200) or GAPDH (1:750) at 4°C overnight. Next, the membrane was incubated with peroxidase-conjugated secondary antibodies (1:1000) for 1 h at 25°C. For analysis of apoptotic markers, we used Apoptosis Western Blot Cocktail assay (Abcam, Cambridge, UK) following the manufacturer´s instructions. The bands were developed using a 3-amino-9-ethyl-carbazole and hydrogen peroxide. Finally, blots were washed and visualized using Azure c600 (Azure Biosystems, Dublin, CA, USA).

### Electrochemical microarray

The obtained cDNA was biotinylated on its 3′ end using the Biotin 3′ End DNA Labeling Kit (Thermo Fisher Scientific) following the manufacturer's instructions. The microarray analyses were performed as previously described by Roth *et al.* [57–59]. For hybridization, Human Cancer 3711 ElectraSense medium density 4 × 2k array slides with 1,609 DNA probes (Custom Array, Bothell, WA, USA) were firstly pre-hybridized for 30 min at 50°C using 6× SSPE (0.9 M NaCl, 60 mM sodium phosphate, 6 mM EDTA), 5× Denhardt´s solution and sonicated salmon sperm DNA (100 μg/ml). Then, the hybridization of biotin-labeled cDNA was performed at 50°C for 18 h in 6× SSPE and salmon sperm DNA (100 μg/ml). Array chips were rinsed with low ionic strength 3× SSPET (3× SSPE, 0.05% Tween-20) and PBST (2× phosphate-buffered saline, pH 7.4, 0.1% Tween-20) to remove weakly bound DNA. Subsequently, array chips were blocked with biotin blocking solution for 15 min. Chips were then incubated for 30 min with poly-horseradish peroxidase-streptavidin (1:1000 in PBS containing 1% bovine serum albumin and 0.05% Tween-20). Next, chips were rinsed three times with biotin wash solution and TMB rinsing solution, followed by incubation with TMB substrate. Measurements were performed using the ElectraSense detection kit (Custom Array). All post-hybridization processing steps were performed at 25°C.

### Wound-healing assay

The cells were pipetted into 6-well plate to reach the confluence ∼80%. After seeding of cells on the bottom of a plate, a pin was used to scratch and remove cells from a discrete area of the confluent monolayer to form a cell-free zone. After that, the cells were re-suspended in a fresh medium. After 48 h, the pictures of cells were taken and compared with pictures obtained at the start-point of experiment. The areas of wounds were analyses and quantified through Olympus IX 71S8F-3 software (Olympus).

### An effect of hMT-3 up-regulation on viability of CDDP-exposed Nbl cells: MTT assay

The viability was assayed using MTT (3-(4,5- dimethylthiazol-2-yl)-2,5-diphenyltetrazolium bromide) assay. Briefly, the suspension of 5,000 cells in 50 μl medium was added to each well of microtiter plates, followed by incubation for 24 h at 37°C with 5% CO_2_ to ensure cell growth. After 24 h treatment, 10 μl of MTT (5 mg/ml in PBS) was added to the cells and the mixture was incubated for 4 h at 37°C. After that, MTT-containing medium was replaced by 100 μl of 99.9% dimethyl sulfoxide and after 5 min incubation, absorbance of the samples was determined at 570 nm using Infinite 200 PRO (Tecan, Männedorf, Switzerland). All analyses were carried out in six replicates. Results are presented as percent of cell viability. Moreover, the viability was also analyzed by Trypan Blue Exclusion (0.4%) and automatically counted with Countess FL II instrument (Invitrogen).

### An effect of hMT-3 up-regulation on proliferation and survival of CDDP-exposed Nbl cells: Clonogenic assay

Cells were seeded in a 6-well plate at a density of 1 × 10^4^ cells per well in a growth medium and incubated for 6 h. Then, the cells were treated with 5 μg/ml CDDP for 48 h. After medium renewal, the cells were incubated for 8 days. Finally, cells were washed with PBS and fixed using 500 μl of 3:1 methanol:acetic acid for 5 min. After fixing, the cells were stained using 500 μl of 0.5% crystal violet in methanol for 15 min. The survival fraction was calculated and related to survival of WT cells.

### Fluorescence microscopy of ROS

Cells were cultivated directly on microscope glass slides (75 × 25 mm, thickness 1 mm, Thermo Fischer Scientific) in Petri dishes. After treatment (5 μg/ml of CDDP, 12 h), microscope glass slides with a monolayer of cells were removed from Petri dishes, rinsed with cultivation and directly used for analysis of ROS using CellROX^TM^ Deep Red Reagent (Thermo Fisher Scientific) according to manufacturer's instructions. For nuclei counterstaining, Hoechst 33258 was employed. Cells were visualized using the EVOS FL Auto Cell Imaging System (Thermo Fisher Scientific).

### Descriptive statistics and exploited bioinformatic tools

For the statistical evaluation of the results, the mean was taken as the measurement of the main tendency, while standard deviation was taken as the dispersion measurement. Differences between groups were analyzed using paired t-test and ANOVA. Unless noted otherwise, the threshold for significance was *p* < 0.05. For analyses, Software Statistica 12 (StatSoft, Tulsa, OK, USA) was employed. The SIMA metabolic pathway was visualized using Kyoto Encyclopedia of Genes and Genomes (KEGG) pathway database (http://www.genome.jp/kegg/), which provides gold standard sets of molecular pathways. The involvement of genes involved in a cellular process was carried out using the Reactome (www.reactome.org).

## SUPPLEMENTARY FIGURES AND TABLES



## Abbreviations

CDDP: *cis*-diamminedichloroplatinum
FBS: fetal bovine serum
GAPDH: glyceraldehyde 3-phosphate dehydrogenase
GFP: green fluorescent protein
hMT-3: human metallothionein-3
MTT: 3-(4,5-dimethythiazol-2-yl)-2
Nbl: neuro-blastoma
OIS: oncogene-induced senescence
PEI: polyethyleneimine
ROS: reactive oxygen species
SQ-RT PCR: semiquantitative reverse transcription polymerase chain reaction
WT: wild-type
