# Supplementary material for: Comparative gene expression profiling of human metallothionein-3 up-regulation in neuroblastoma cells and its impact on susceptibility to cisplatin
Source: Oncotarget. 2017 Dec 16;9(4):4427–39. doi: 10.18632/oncotarget.23333 (PMC5796984; doi:10.18632/oncotarget.23333)
Supplement: Supplementary file 1 [file oncotarget-09-4427-s001.pdf]

Comparative gene expression profiling of human metallothionein-3 up-regulation in neuroblastoma cells and its impact on susceptibility to cisplatin

SUPPLEMENTARY MATERIALS

REFERENCES

1. Amoroso L, Erminio G, Makin G, Pearson AD, Brock P, Valteau-Couanet D, Castel V, Pasquet M, Laureys G, Thomas C, Luksch R, Ladenstein R, Haupt R, Garaventa A; SIOPEN Group. Topotecan-Vincristine-Doxorubicin in Stage 4 High Risk Neuroblastoma Patients Failing to Achieve a Complete Metastatic Response to Rapid COJEC - a SIOPEN Study. Cancer Res Treat. 2017 Mar 21. https://doi.org/10.4143/crt.2016.511.

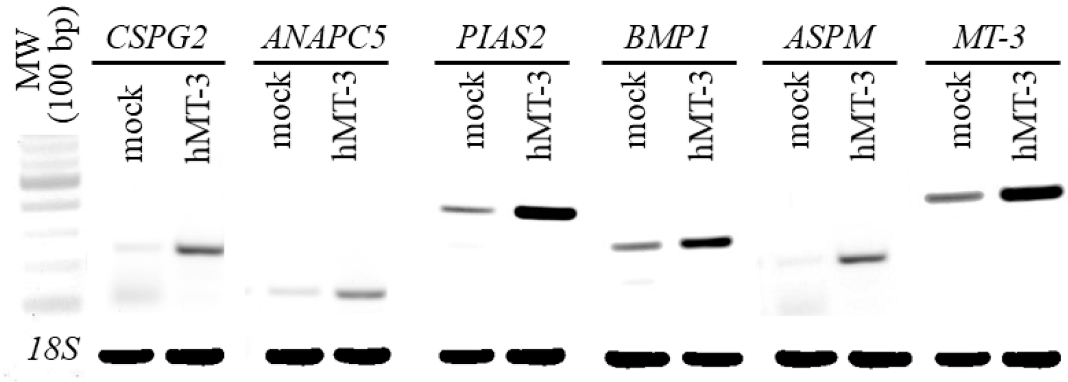

Supplementary Figure 1: SQ-RT-PCR validation of microarray results. We validated genes with the expression 2.5-fold stronger than in mock cells. To adjust the amount of transcribed cDNA, *18S* ribosomal RNA was selected as an internal control. For evaluation of differences in gene expression between *hMT-3* and mock, 10 µL of each SQ-RT-PCR product was electrophoresed on a 2.0% agarose gel and stained with ethidium bromide.

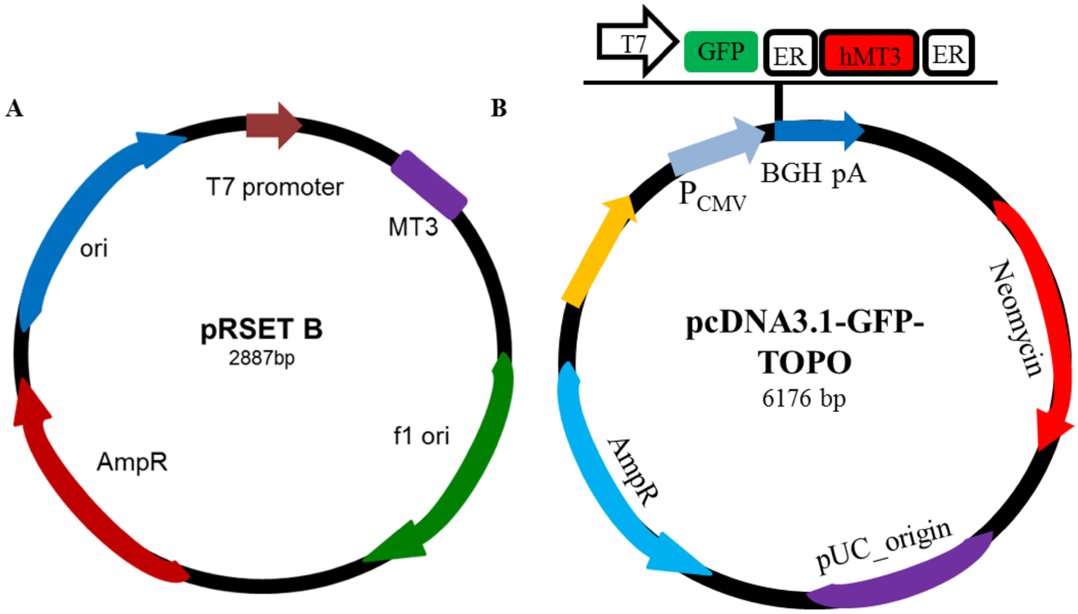

C

ATGGACCCTGAGACCTGCCCCTGCCCTTCTGGTGGCTCCTGCACCTGCGCGGA

CTCCTGCAAGTGCGAGGGATGCAAATGCACCTCCTGCAAGAAGAGCTGCTGCT

CCTGCTGCCCTGCGGAGTGTGAGAAGTGTGCCAAGGACTGTGTGTGCAAAGG

CGGAGAGGCAGCTGAGGCAGAAGCAGAGAAGTGCAGCTGCTGCCAG**TGA**

Supplementary Figure 2: The plasmid maps showing the features of (**A**) hMT3-pRSET-B and (**B**) pcDNA3.1-GFP-TOPO. (**C**) Nucleotide sequences of *hMT3* cloned in pcDNA3.1-GFP-TOPO. AmpR: ampicillin resistance, T7: T7 promoter/priming. P_{CMV}: cytomegalovirus promoter, GFP: green fluorescence protein, ER: multiple cloning site.

Supplementary Table 1: Data on Nbl specimens obtained from patients in 2003-2013 (1–23), included are also data on *hMT-3* expression in control non-malignant cell lines derived from adrenal cortex

No.	Alive	*N-Myc* amplification	Chemotherapy	Stage	*hMT-3*
1	Y	N	Y	4	13.950
2	Y	Y	N	3	11.095
3	Y	Y	N	2	14.480
4	N	N	N	2	13.620
5	N	G	Y	4	14.500
6	N	Y	N	4	12.630
7	N	N	N	4	13.065
8	Y	Y	N	4	13.155
9	Y	Y	N	4	13.915
10	Y	Y	N	4	12.550
11	Y	Y	N	4	14.840
12	N	Y	N	4	15.235
13	N	Y	Y	4	15.535
14	Y	Y	N	4	15.825
15	Y	Y	N	3	15.085
16	N	N	N	4	15.245
17	N	Y	N	4	12.875
18	N	N	Y	4	16.120
19	N	Y	N	4	14.455
20	N	Y	N	4	13.935
21	N	N	N	4	16.720
22	Y	Y	N	4	19.245
23	Y	Y	N	4	17.385
HadCC	-	-	-	-	1.256
EJG	-	-	-	-	0.699
SBAC	-	-	-	-	2.365

Y- yes, N- no.; *N-Myc* amplification - Y = over 4-fold increase in the *N-Myc* signal number in relation to the number of chromosomes 2; G = *N-Myc* gain- up to 4-fold excess of copies of *N-Myc* in relation to the number of chromosomes 2, ; N = no amplification – number of *N-Myc* copies is equal to the number of chromosomes 2; chemotherapy - N = sample was collected before starting the chemotherapy, Y = sample was collected during or after chemotherapy according to study HR-NBL-1/SIOPEN; stage - clinical stage according to INSS Staging [1]. *hMT-3* is presented as relative fold gene expression ($2^{-\Delta\Delta CT}$), *18S* ribosomal was used as housekeeping gene.

Supplementary Table 2: List of primers employed for validation of selected microarray results using semiquantitative PCR

Gene	Symbol	Primer pair (5′-3′)*	T_m
ARP2 anaphase promoting complex subunit 5	*ANAPC5*	CGTGTGTCTCCAGCACTGTT<br>TCCTTTAGGGCATCCATCAG	60.0
Cyclin dependent kinase inhibitor 2B 6	*CDKN2B*	TAGTGGAGAAGGTGCGACAG<br>GGTGAGAGTGGCAGGGTCT	60.0
Glutathione S-transferase mu 3	*GSTM3*	TAATGGATTTCCGCACACAA<br>CCTTCAGGTTTGGGAACTCA	60.0
Caspase 4	*CASP4*	TGAAGGACAAACCCAAGGTC<br>TCTCTCCAGGACACGTTGTG	59.9
DnaJ heat shock protein family (Hsp40) member B6	*DNAJB6*	AAGCAAGTAGCGGAGGCATA<br>AATGAAAATGGGTCCCTTCC	60.0
Metallothionein 3	*hMT3*	ATGGACCCTGAGACCTGCCC<br>TCACTGGCAGCAGCTGCACT	61.0
18S ribosomal RNA	*18S*	CGGCTACCACATCCAAGGAA<br>GCTGGAATTACCGCGGCTGC	60.0

*Upper and lower sequences represent forward and reverse primers, respectively; T_m is melting temperature of specific product.
